# Viscodelamination of Localized Retrolental Plaques During Lens-Sparing Vitrectomy in Eyes With Pediatric Tractional Vitreoretinopathy

**DOI:** 10.1097/IAE.0000000000002834

**Published:** 2020-06-08

**Authors:** Jiao Lyu, Qi Zhang, Peiquan Zhao

**Affiliations:** Department of Ophthalmology, Xinhua Hospital, School of Medicine, Shanghai Jiao Tong University, Shanghai, China.

**Keywords:** viscoelastic, delamination, retrolental membrane, vitrectomy, pediatric retinopathy

## Abstract

To report viscodelamination of localized retrolental plaques during lens-sparing vitrectomy in eyes with pediatric tractional vitreoretinopathy.

**Methods:**

Viscodelamination of retrolental plaques was performed during 23-gauge lens-sparing vitrectomy in 11 eyes from 11 children with a median age (range) of 12 (4–58) months. There were five eyes with Stage 4 familial exudative vitreoretinopathy, one eye with Stage 4B retinopathy of prematurity, and five eyes with anterior–posterior persistent fetal vasculature syndrome. Retrolental plaques were separated from the posterior lens capsule by the tension of viscoelastic injection in the Berger's space.

**Results:**

In 10 of 11 eyes, retrolental plaques were removed from posterior surface of lens without capsular tearing. During the 12-month to 18-month follow-up period, lens clarity along the visual axis was retained in 5 eyes with familial exudative vitreoretinopathy, one eye with retinopathy of prematurity, and 2 of 5 eyes with persistent fetal vasculature. Atraumatic viscodelamination was associated with retrolental plaques that did not incorporate into the posterior lens capsule. Retinal dragging was reversed in all 11 eyes.

**Conclusion:**

Viscodelamination may be effective for the dissection of retrolental plaques related to pediatric vitreoretinopathy in selected cases.

## Abstract 2

Supplemental Digital Content is Available in the Text.

Unregressed embryonic hyaloid tissue in persistent fetal vasculature (PFV) syndrome or assembly of cell migration and extracellular matrix in advanced familial exudative vitreoretinopathy (FEVR) or retinopathy of prematurity (ROP) can induce retrolental proliferation that extends from the retina to the anterior hyaloid membrane (AHM) and posterior lens capsule.^1–3^ To preserve the clarity of the crystalline lens and to release tractional vectors, methods have demonstrated the dissection of localized retrolental plaques in lens-sparing vitrectomy (LSV) for PFV, advanced ROP, and FEVR.^2,4–7^ Mechanically stripping of proliferative tissue from the posterior lens surface during LSV frequently resulted in progressive cataract.^2,4^ Lens-sparing transection of retrolental plaque at its posterior aspect using a microvitreoretinal blade or microscissors can reduce injury to the overlying lens capsule. However, transection methods risk inadvertent penetration of the anteriorly inserted retina in the retrolental tissue.^6–8^ Still, postoperative residual retrolental tissue obscures vision. The efficacy of elimination of the localized retrolental plaques from posterior lens capsule during LSV for pediatric tractional retinopathies has not been published.

The Berger's space between the AHM and the posterior lens capsule can be dissected with viscoelastic or fluid.^9–11^ Accordingly, we attempted viscoelastic delamination of retrolental plaques by exploring the Berger's space between AHM and posterior lens capsule, during LSV for FEVR, ROP, or PFV. We observed that entire removal of retrolental plaques without evident damage to the lens can be achieved in selected cases by the technique.

## Patients and Methods

The retrospective case series included consecutive patients who had tractional vitreoretinopathies and received 23-gauge LSV surgery and viscodelamination of retrolental tissues in the affected eyes, at Shanghai Jiaotong University, Medical School, Xinhua Hospital, between August 2017 and August 2018 (Table 1). All procedures were conducted in accordance with the Declaration of Helsinki and approved by the ethics committee of Xinhua Hospital. Informed consent for all surgical procedures was obtained from the legal guardian of participants.

**Table 1.** Characteristics of Eyes With Pediatric Vitreoretinopathy Undergoing Viscodelamination and Lens-Sparing Vitrectomy

Pt	Sex	Age at Surgery, months	Etiologies	Eye	Preoperative and Intraoperative Observation						Postoperative Outcomes		
					Lens	Fundus	Characteristics of Retrolental Plaques				FU	Lens	Fundus
							Adhesion Plane	Morphology	Location	Largest Diameter, (mm)			
1	M	36	FEVR (4B)	R	Clear	Temporal retinal folds, TRD	On AHM, with pinpoint lens–plaque adhesion	Stellate membrane	Central	1	18	Clear	Partly resolved TRD
2	M	12	FEVR (4B)	L	Clear	Temporal retinal folds, TRD	On AHM, with pinpoint lens–plaque adhesion	Wedge-shaped membrane	Central	3	18	Clear	Partly resolved TRD
3	M	5	FEVR (4B)	L	Mild capsular opacity in periphery	Temporal retinal folds, TRD	On AHM, with focal lens–plaque adhesion in periphery	Stellate membrane	Central	3	18	No change	Resolved TRD
4	M	4	FEVR (4B)	R	Mild capsular opacity in periphery	Temporal retinal folds, TRD	On AHM, with focal lens–plaque adhesion in periphery	Wedge-shaped membrane	Pericentral, temporal	3	12	No change	Resolved TRD
5	M	58	FEVR (4B)	L	Clear	Temporal retinal folds, TRD	On AHM, with pinpoint lens–plaque adhesion	Wedge-shaped membrane with radial fibrosis	Peripheral, temporal	2	12	Clear	Resolved TRD
6	M	6	ROP (4B)	L	Clear	Nasal TRD involving posterior pole	On AHM, with focal lens–plaque adhesion	Irregular shaped fibrovascular nodule, with blood clot	Peripheral, inferonasal	2	12	Clear	Flat posterior pole, nasal fibrosis
7	M	36	A-P PFV	R	Clear	Parapapillary TRD and stalk	On AHM, with pinpoint lens–plaque adhesion	Brittle-star membrane	Pericentral, nasal	2	12	Clear	Attached retina
8	F	7	A-P PFV	R	Axial capsular opacity	Parapapillary TRD and stalk	On posterior lens capsule	Brittle-star membrane	Central	3	12	Significant subcapsular cataract in 3 months, lensectomy, aphakia	Attached retina
9	M	18	A-P PFV	L	Axial capsular opacity	Parapapillary TRD and stalk	On posterior lens capsule	Brittle-star membrane	Central, nasal	3	12	Mild subcapsular opacification	Attached retina
10	M	12	A-P PFV	R	Capsular tear during delamination	Parapapillary stalk, epiretinal membrane	On posterior lens capsule	Brittle-star membrane, perfused hyaloid artery	Central	2	12	Aphakia	Flat retina
11	M	48	A-P PFV	R	Clear	Parapapillary TRD and stalk after a 10-month observation	On AHM, with focal lens–plaque adhesion	Brittle-star membrane	Peripheral, inferonasal	2	12	Clear	Attached retina

A-P, anterior–posterior; F, female; FU, follow-up; M, male.

Surgeries were performed to clear the visual media and unravel retinal dragging. Included were eyes with localized retrolental tissues and focal tractional retinal detachment (TRD) or distortion in the posterior pole. Excluded were eyes with marked cataract, obvious deficiency of lens capsule (tear, opacity, deformation), lens luxation, elongated ciliary process, extensive anterior proliferation, and retinal detachment, ause a lensectomy/vitrectomy was indicated.

**Fig. 1.**
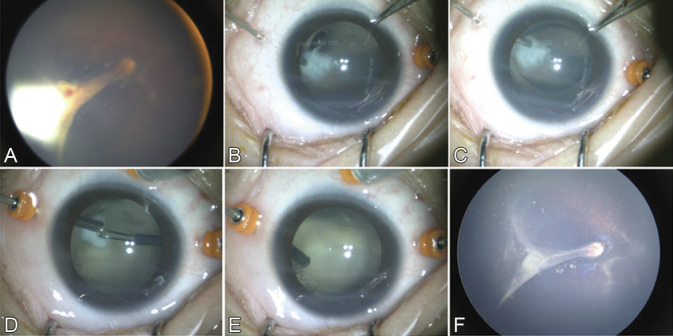
Viscodelamination of a retrolental plaque during lens sparing vitrectomy in the right eye of Patient 4 with Stage 4B FEVR. **A.** The eye showed a temporal retinal fold and fibrovascular proliferation attaching to lens. **B.** A 26-gauge cannula through one sclerotomy was used to gently lift the retrolental plaque from the posterior lens capsule and to inject viscoelastic into the Berger's space. **C.** The plaque was separated from the lens capsule by the tension of the viscoelastic injection. The plaque was found to be on the AHM with focal adhesion to the posterior lens capsule. **D.** The plaque was excised bimanually with vitreoretinal forceps and a vitreous cutter. **E.** The plaque was entirely removed and lens clarity along the visual axis was preserved. **F.** Fundus examination by wide-angle fundus imaging system RetCam (clarity medical systems, Pleasanton, CA), 12-month postoperatively, showed a clear media through clear lens and improved posterior structure.

Surgical technique (Figure 1; video, Supplemental Digital Content 1, http://links.lww.com/IAE/B229).

The retrolental plaque was firstly explored with illumination under an operating microscope for its location, extent, and tractional role on the adjacent tissues. Sclerotomies for a 23-gauge LSV surgery were then made where it was advantageous to access the retrolental plaque without damage to the retina. Viscodelamination was performed before introduction of infusion. One trocar cannula in the sclerotomy was transiently removed to host an angled 26-gauge cannula. A 26-gauge cannula entered the sclerotomy, gently lifted the edge of the retrolental plaque from the posterior lens capsule with its blunt tip, and continually injected cohesive viscoelastic (Aiwei, Bausch and Lomb; Shandong Furuida Co., Ltd., Shandong, China) into the Berger's space. The viscoelastic dispersed in the Berger's space and above the retrolental plaque. The plaque was separated from the posterior lens capsule by the tension of the viscoelastic and then excised by vitreous cutter. The amount of viscoelastic used was about 0.1 mL to 0.3 mL. The opposite trocar cannula was unplugged in case of hypertension due to viscoelastic tamponade. Viscodelamination adjacent to Wieger's ligament was done with caution to preserve the zonules. For firmly adhering plaques, viscodelamination was performed in combination with peeling and cutting using vitreoretinal forceps and a vitreous cutter. Tractional vitreoretinopathies were addressed in the LSV surgery, followed with fluid–air exchange. The patients were in a face-down position for 7 days. Topical prednisolone acetate, fluoroquinolone, and tropicamide were each administered four times daily for two weeks postoperatively. The patients were followed up weekly, then monthly, and bimonthly for at least 12 months. Lens opacity was considered significant if it hampered the view of the posterior segment by indirect ophthalmoscopy.

## Results

Eleven eyes of 11 patients received viscodelamination of retrolental plaques during 23-gauge LSV surgery. There were eight central or pericentral and three peripheral retrolental plaques with a largest diameter from 1 mm to 3 mm, in five eyes with Stage 4B FEVR, one eye with Stage 4B ROP, and five eyes with anterior–posterior PFV. Two eyes with FEVR and two eyes with PFV had mild lens opacity adjacent to retrolental plaques. One eye with PFV (Patient 11) and an inferonasal plaque had a progressive TRD involving the macula during a 10-month observation before surgery.

**Fig. 2.**
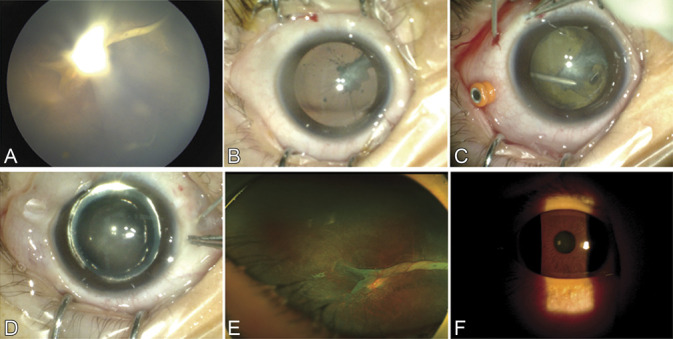
Viscodelamination of a retrolental plaque during lens sparing vitrectomy in the right eye of Patient 7 with combined type of PFV. **A** and **B.** The eye showed a hyaloid fibrovascular stalk extending from the optic disk to the posterior lens. The eye showed parapapillary TRD. **C.** Viscodelamination indicated a retrolental plaque on the AHM with pinpoint adhesion to posterior lens capsule. **D.** The retrolental plaque was removed and lens clarity was preserved after fluid–air exchange. **E.** Fundus examination by ultrawide-field scanning laser ophthalmoscope (UWF SLO, Optos PLC; Dunfermline, Scotland, United Kingdom) imaging 3-month postoperatively showed an improved posterior structure. **F.** Eight-month postoperative examination showed a clear lens.

Retrolental plaques were delaminated from the posterior lens capsule without an apparent capsular tear in 10 (91%) of the 11 eyes. After a 12-month to 18month follow-up, lens clarity along the visual axis was maintained in eight eyes (73%), including 5 eyes with FEVR (Figure 1), one eye with ROP, and 2 of 5 eyes with PFV (Figure 2). Reversal of retinal dragging was observed in all eyes (Table 1).

In the eight eyes without a visually significant cataract after surgery, intraoperative observation and atraumatic delamination in the Berger's space revealed that the retrolental plaques were on the AHM with focal attachment to the posterior lens capsule. Preexisting mild capsular opacity in two eyes with FEVR did not significantly progress after delamination. Visual acuity available in one eye with PFV (Patient 11) improved from counting fingers to 20/500 12 months postoperatively. The other three eyes with PFV had central plaques invading the posterior lens capsule. A capsular tear occurred after viscodissection in the Berger's space near the perfused hyaloid arteries in the plaque in one eye (Patient 10), leading to a concomitant lensectomy. Progression of preexisting mild capsular opacity was observed in two eyes with PFV 3 months postoperatively; one eye (Patient 8) with significant cataract received a secondary lensectomy.

## Discussion

The current report explored the Berger's space as a surgical plane for viscodelamination of retrolental plaques in eyes with advanced FEVR, ROP, and PFV during LSV surgery. The efficacy of delamination of retrolental plaques depends on how retrolental tissues invade the Berger's space and affect the posterior lens capsule.^9,11^ Retrolental plaques are formed secondarily in eyes with FEVR or ROP^1^ as these plaques adhere to the AHM before invading the Berger's space and posterior lens capsule. Thus, it was effective to inject viscoelastic from the adjacent Berger's space into the lens–plaque adhesion for delamination and to respect the posterior lens capsule. Efficacy of viscodelamination seemed less relative to the central or noncentral location of plaques.

In eyes with PFV, viscodelamination may be unharmful when the retrolental plaques were mostly retrograde to the AHM.^12,13^ Affected eyes of Patients 7 and 11 showed retrolental plaques on the AHM with pinpoint adhesion to the posterior lens capsule and consequently they developed no obvious lenticular opacification after the surgery. It is noteworthy that Patient 11 received LSV due to a progressive TRD involving the macula. Removal of the inferonasal retrolental plaque and epiretinal tractional force yielded good anatomical and visual improvement postoperatively. However, viscodelamination is invasive for treating retrolental stalks incorporated into the posterior lens capsule, as frequently observed in eyes with PFV. Unregressed fetal hyaloid tissues may impair the formation of Berger's space and compromise the integrity of lens capsule. Lenticular changes, such as fenestrations on the posterior lens capsule, thinning, or deformation of the lens capsule, may exist in proximity to the hyaloid artery system in Cloquet's canal.^13–15^ The undetected defect of the posterior lens capsule near the plaque may account for the capsular rupture during delamination in the eye of Patient 10 and progressive cataract after delamination in the eyes of Patients 8 and 9. Such complications of viscodelamination were typically with central plaques toping at the Cloquet's canal.

Clearance of the retrolental tissues enhanced surgical visualization in LSV. Removal of contractile retrolental tissues attached to the retina allowed release of the anteroposterior vector. Thus, it encouraged anatomical recovery with resolution of TRD in all the cases.

Issues pertaining to the technique require attention. The technique is meant for localized retrolental adhesion but not for extensive lesions. In the presence of strong lens–plaque adhesion or adhesion involving Wieger's ligament, any forceful viscodelamination is prohibited because it may induce a capsular tear, anterior retinal dialysis, or zonular dehiscence. One may also combine viscodelamination with partial transection on the premise that all the traction is sufficiently released and that transection does not risk penetrating the anteriorly inserted retina. In addition, because of the limited cases in the current study, all appropriate types of retrolental plaques for a delamination during LSV have not been investigated. Short-term observation is valuable to demonstrate the early-phase status of the lens after surgery. Long-term stability of the lens and retina should be confirmed in a prolonged study.

In conclusion, viscodelamination may be an effective surgical alternative to the dissection of retrolental plaques related to pediatric vitreoretinopathies in selected cases undergoing LSV. The adhesion plane of the retrolental plaques and underlying etiologies affect the efficacy of delamination and the postoperative status of the lens.

## Supplementary Material
